# Recent Progress in Stimuli-Responsive Antimicrobial Electrospun Nanofibers

**DOI:** 10.3390/polym15214299

**Published:** 2023-11-01

**Authors:** Luiza A. Mercante, Kelcilene B. R. Teodoro, Danilo M. dos Santos, Francisco V. dos Santos, Camilo A. S. Ballesteros, Tian Ju, Gareth R. Williams, Daniel S. Correa

**Affiliations:** 1 Institute of Chemistry, Federal University of Bahia (UFBA), Salvador 40170-280, BA, Brazil; 2 Nanotechnology National Laboratory for Agriculture (LNNA), Embrapa Instrumentação, São Carlos 13560-970, SP, Brazil; kbr.teodoro@gmail.com (K.B.R.T.); martinsdanilo.9@gmail.com (D.M.d.S.); francisco_santos@usp.br (F.V.d.S.); 3 Department of Materials Engineering, São Carlos School of Engineering, University of São Paulo, São Carlos 13563-120, SP, Brazil; 4 Bachelor in Natural Sciences and Environmental Education, Pedagogical and Technological University of Colombia (UPTC), Tunja 150003, Colombia; carturosuarezb@gmail.com; 5 UCL School of Pharmacy, University College London, 29-39 Brunswick Square, London WC1N 1AX, UK; tian.ju.19@ucl.ac.uk (T.J.); g.williams@ucl.ac.uk (G.R.W.)

**Keywords:** electrospinning, electrospun nanofibers, stimuli-responsive, smart materials, light-responsive, pH-responsive, thermo-responsive, antimicrobial, antibacterial

## Abstract

Electrospun nanofibrous membranes have garnered significant attention in antimicrobial applications, owing to their intricate three-dimensional network that confers an interconnected porous structure, high specific surface area, and tunable physicochemical properties, as well as their notable capacity for loading and sustained release of antimicrobial agents. Tailoring polymer or hybrid-based nanofibrous membranes with stimuli-responsive characteristics further enhances their versatility, enabling them to exhibit broad-spectrum or specific activity against diverse microorganisms. In this review, we elucidate the pivotal advancements achieved in the realm of stimuli-responsive antimicrobial electrospun nanofibers operating by light, temperature, pH, humidity, and electric field, among others. We provide a concise introduction to the strategies employed to design smart electrospun nanofibers with antimicrobial properties. The core section of our review spotlights recent progress in electrospun nanofiber-based systems triggered by single- and multi-stimuli. Within each stimulus category, we explore recent examples of nanofibers based on different polymers and antimicrobial agents. Finally, we delve into the constraints and future directions of stimuli-responsive nanofibrous materials, paving the way for their wider application spectrum and catalyzing progress toward industrial utilization.

## 1. Introduction

The threat that microorganisms pose to human health, the environment, and food safety has become a serious concern in recent years, and microbial pathogens are the major contributors to illness and death globally [1,2,3]. Conventional methods to kill or inhibit microorganism growth include antimicrobial drugs and a wide selection of nonpharmaceutical chemicals [4,5,6]. Nonetheless, the extensive and widespread overprescription of antimicrobial drugs and their substantial release into the environment has led to the emergence of drug-resistant strains [7,8,9,10,11,12]. For instance, according to the World Health Organization (WHO), antimicrobial resistance (AMR) is one of the top ten most urgent global health threats [7,13]. The number of deaths associated with AMR is expected to reach 10 million per year worldwide by 2050 [11]. Moreover, the global economic loss caused by AMR is estimated to reach approximately $100 trillion by 2050 [14]. Therefore, in the last few years, there has been increasing attention paid towards the development of alternative strategies for the use of antibiotics.

Recent advances in nanotechnology and material science have opened promising avenues for developing functional antimicrobial materials that are able to fight against pathogenic microorganisms, including bacteria, fungi, and viruses [15,16]. Among these emerging strategies, stimuli-responsive materials have garnered considerable attention as a highly intriguing approach to combating microbial threats [4,17,18,19,20]. A stimuli-responsive material, also known as a smart material, refers to a material capable of sensing the surrounding environment and subsequently modifying its intrinsic chemical or physical properties in response to one or more triggering stimuli [17,18,19]. Depending on the material type/structure, the triggering stimuli can be physical (e.g., temperature, light, magnetic field, electric interaction), chemical (e.g., pH, redox potential), or biological (e.g., enzymatic) [8]. In response, smart materials can selectively and efficiently change their physical and/or chemical properties, thereby enabling them to dynamically control the release or exposure of antimicrobial agents [17,18]. This precise spatial and temporal control over the microbial environment is particularly advantageous for targeted and effective antimicrobial therapy [4].

Stimuli-responsive antimicrobial materials are typically prepared by combining a stimuli-responsive component (usually a smart polymer) with an antimicrobial agent, and can be engineered in various forms, including thin films [21,22], nano/microparticles [23,24], micelles [25], and nanofibrous membranes [26,27,28]. Recently, electrospinning has emerged as one of the most effective methods for fabricating stimuli-responsive antimicrobial nanofibrous membranes. In this process a high voltage is applied to a droplet of polymer solution or melt, creating an electrically charged jet of material. The repulsion between the charges in the jet causes it to elongate and narrow down into a fine filament as it travels toward a collector. As the solvent evaporates or the polymer solidifies during flight, a continuous, interconnected network of fibers is formed on the collector, resulting in a nonwoven mesh-like structure known as an electrospun nanofibrous membrane [29,30,31]. The basic electrospinning setup includes a high-voltage power supply, a syringe connected to a spinneret (dispensing needle), and a conductive collector. The final fiber morphology and properties (e.g., diameter, surface roughness, porosity) are determined by the integrative action of several experimental parameters, which can be classified into three categories: solution parameters, process parameters, and environmental parameters [32]. By manipulating these factors materials with high surface area, fine structural features, and diverse functional properties can be fabricated in a straightforward and cost-effective way [33,34,35,36,37]. A detailed discussion of these parameters can be found elsewhere [38,39,40,41].

The high surface area and porosity of electrospun nanofibrous materials are beneficial for encapsulating bioactive compounds, leading to high efficiency in numerous applications, including tissue engineering [42,43], drug delivery [44,45], wound dressings [46,47], food packaging [46,47] and filtration [46,47]. Furthermore, the electrospinning process can be carried out at room temperature, preventing the volatilization or degradation of chemical compounds, and thereby avoiding loss of biocide efficiency [48].

**Scheme 1:**
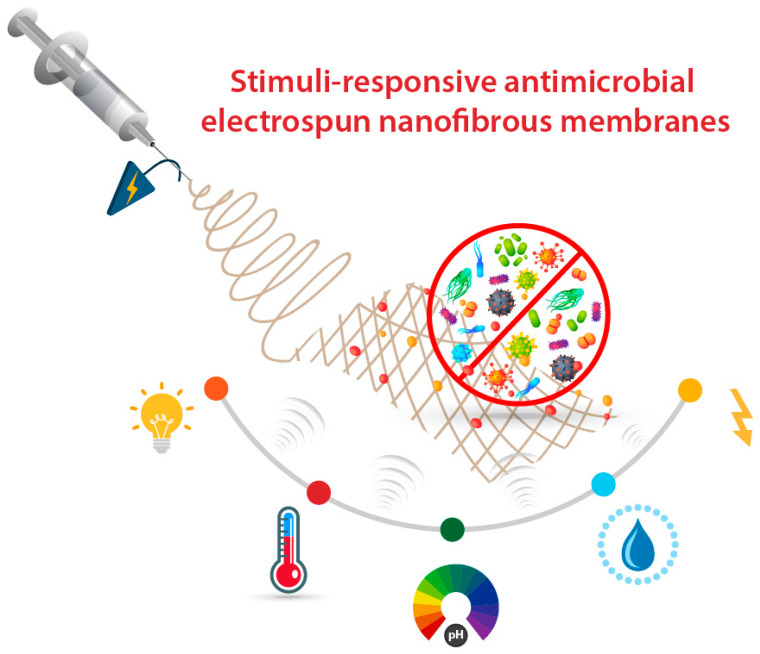
Schematic illustration of stimuli-responsive antimicrobial electrospun nanofibrous membrane: when a stimulus is applied (e.g., light, temperature, pH, humidity, electric field), the nanofibers respond by triggering the antimicrobial response.

Although some interesting reviews on stimuli-responsive antimicrobial electrospun nanofibers are available in the literature, they are mainly focused on another type of application [28,29,49] or, instead, on a specific type of stimulus [33,50,51,52]. Herein, we aim to review recent advances in stimuli-responsive antimicrobial materials based on electrospun nanofibers, as illustrated in Scheme 1. Initially, we provide an overview of different strategies for developing smart electrospun nanofibrous membranes with antimicrobial properties. We then present the potential use of different systems in which one or multiple stimuli induce an antimicrobial response. Finally, the challenges and outlook are outlined to promote further development of smart electrospun materials.

## 2. Design of Smart Electrospun Nanofibers with Antimicrobial Properties

**Figure 1:**
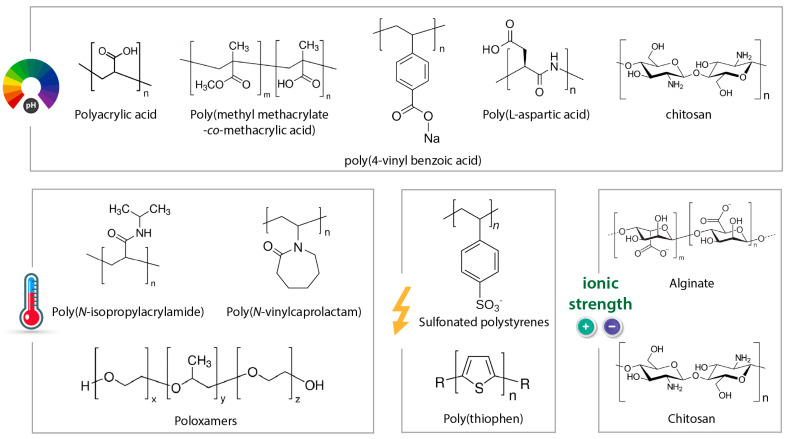
Chemical structures of some typical stimuli-responsive polymers.

Smart materials should have a fast-switching mechanism or one that maintains a suitable environment fit for the intended purpose [34,53,54]. The material properties in each switched state must be distinctly different for an effect to be observed. To achieve this goal, there is a need for fundamental knowledge of the material’s properties/composition. In the case of polymers employed in the construction of smart nanofibrous materials, they can be designed to respond to various stimuli, such as temperature, pH, and humidity, as shown in Figure 1. If the polymer used to generate the nanofibrous membrane does not respond to a stimulus, additional components (molecules or nanomaterials) could be added to produce a nanofibrous membrane with the desired response (e.g., electrical, biological, optical) [4,55].

Different agents can be used to impart antimicrobial properties to the nanofibrous membranes [56]. Incorporating classical antimicrobials, such as antibiotics, antifungals, and antivirals, is the most common strategy to achieve nanofibrous membranes with active antimicrobial function [56]. Naturally sourced antimicrobial agents (e.g., plant extracts, biopolymers) have also shown a great potential to control and inhibit the growth of microorganisms [56,57]. Generally, the active components of plant extracts inhibit microorganisms through the disturbance of the cytoplasmic membrane, disrupting the proton motive force, electron flow, active transport, and/or inhibition of protein synthesis [56]. Certain types of metals (e.g., Ag, Cu, Au, Pd) and metal-oxide nanomaterials (e.g., ZnO, TiO_2_, CuO, CeO_2_) [10,58,59], MXenes [60,61,62], bioglass [63,64,65], Fe_3_O_4_ [66,67,68], and some metal organic frameworks (MOFs) [5,69,70,71,72] are also known to have broad-spectrum antimicrobial activity. Similar to conventional drugs, antimicrobial nanomaterials act through one or more mechanisms to inhibit microbial growth or kill microorganisms [73].

These antimicrobial agents could be incorporated into the nanofibers via a range of different strategies [74,75]. The most common method is the blending approach, where the antimicrobial agent is dissolved or dispersed throughout the polymer solution before electrospinning [76,77]. This method subjects the active molecule to high voltages and organic solvents that may reduce or destroy its biocide activity [78]. The bioactivity is often better retained when the therapeutic is encapsulated via emulsion or coaxial electrospinning [79]. In the emulsion electrospinning approach, the polymer and active agent are dissolved in immiscible solvents before the electrospinning process [80]. This method ensures double protection for the antimicrobial agent when placed inside another carrier system, such as liposomes, nanoparticles, or nanocapsules, in an emulsion droplet [80]. Dendrimers, a class of highly branched macromolecules, can also be applied to encapsulate antimicrobial agents, especially via non-covalent interactions (e.g., electrostatic, hydrophobic, hydrogen-bond) [81,82,83,84]. In this regard, poly(amidoamine) (PAMAM) is the most widely recognized dendrimer used to develop dendrimer-modified nanofibrous membranes aiming at antimicrobial applications [81]. Coaxial electrospinning involves two syringes and a spinneret comprising two concentric nested needles. It allows the formation of nanofibers that have core/shell structures [79,85,86]. In this case, the core solution is usually an aqueous solution containing hydrophilic therapeutics in order to improve stability or even extend release duration [87,88,89]. Finally, the active molecules can also be incorporated after the electrospinning process by modifying the surface of the nanofibers. However, surface immobilization may directly expose sensitive molecules to potentially harmful microenvironments, reducing the bioactivity of the therapeutic cargo [90].

The use of nanofibers with Janus asymmetric architecture [91,92] has also been reported as a promising strategy in the development of smart antimicrobial materials [93]. For instance, nanofibers based on poly(lactic acid) (PLA)/polyacrylonitrile (PAN) and incorporated with phenol red and oxaline were demonstrated to be responsive to pH changes. In addition, in vitro (using *Staphylococcus aureus* (*S. aureus*)) and in vivo (rats) tests have shown that electrospun materials are capable of monitoring the wound state via changes in the medium pH (caused by the infection), and still inhibit bacterial growth [93]. In another study, self-assembled and photo-responsive electrospun membranes based on polyvinylidene fluoride (PVDF)/bismuth titanate (Bi_4_Ti_3_O_12_)/MXene (Ti_3_C_2_T_x_) were also able to prevent pathogenic infections [94]. The results demonstrated that electron transfer from Bi_4_Ti_3_O_12_ to Ti_3_C_2_T_x_ was possible, which enhanced the material’s light sensitivity and conferred excellent antibacterial activity (99%) against *Escherichia coli* (*E. coli*) and *S. aureus*.

**Figure 2:**
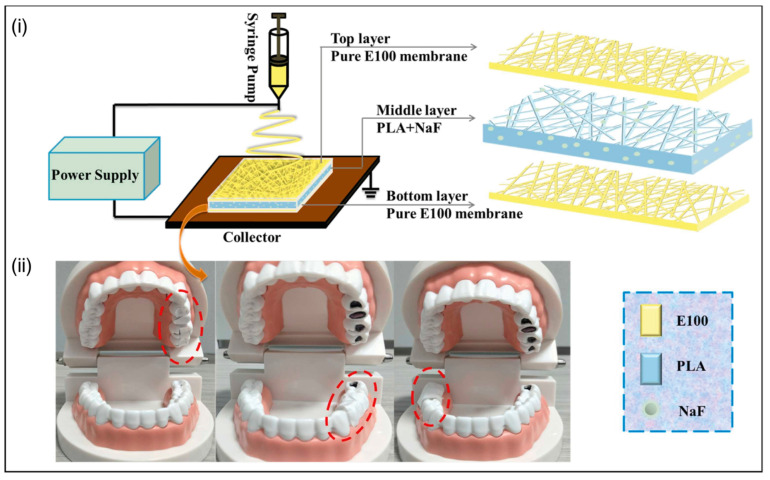
(**i**) Manufacturing process of toothpaste based on electrospun nanofibers of PLA/E100/NaF. These are responsive to pH changes and have a sandwich structure. (**ii**) Images of toothpaste fixation in a caries model to demonstrate the functionality of the nanofibers and the location (dashed area) where the responsive nanofibrous dental paste was applied. Reprinted with permission from reference [96]. Copyright 2023 Elsevier.

A thermoresponsive sandwich structure was engineered using a poly(N-isopropyl acrylamide) hydrogel containing silver nanocubes and electrospun poly(ɛ-caprolactone) (PCL)/poly(ethylene oxide) (PEO) nanofibers [95]. In vitro tests carried out with *S. aureus* revealed that, after 4 h of incubation, the platform enriched with silver nanocubes (20 wt%) successfully eliminated 99.9% of the bacteria. In another study, a pH-responsive nanofiber-based toothpaste, employing a sandwich structure comprising PLA/methylmethacrylate IV (E100) containing sodium fluoride (NaF), as illustrated in Figure 2, was developed [96]. In vitro experimentation demonstrated the system’s efficacy in inhibiting the growth of *Streptococcus mutans* (*S. mutans*) bacteria.

Regarding the stimuli sensitivity of electrospun materials, studies have shown that the nanofiber properties (e.g., size, porosity, roughness, diameter, and surface chemistry) can affect their response towards several stimuli, including heat, light, pressure, or humidity [97]. For example, the porous microstructure (5.45 m^2^/g) of electrospun membranes, based on cellulose diacetate incorporated with protoporphyrin IX and potassium iodide, favored interactions between the pathogens and reactive oxygen species (ROS) generated after laser irradiation (λ ≥ 420 nm, 66 mW/cm^2^ for 30 min), resulting in an antibacterial efficiency of 99% against *E. coli* and *S. aureus* [98].

The combination of 3D printing technologies and electrospinning has allowed the construction of highly customized, versatile, and functional platforms aimed at different applications [99,100,101,102,103]. To this end, electrospun nanofibers and 3D printing were combined for the manufacture of light-responsive face masks containing gold nanoparticles with excellent antimicrobial properties [102].

## 3. Stimuli-Responsive Electrospun Nanofibers

The convergence of polymer science and nanotechnology has created a synergistic opportunity for designing nanofibrous membranes with stimuli-responsive antimicrobial properties. To date, diverse smart electrospun nanofibers with antimicrobial properties have been successfully prepared using different polymers and biocide agents. Some illustrative examples are summarized in Table 1, and operate under a range of different (physical, chemical, or biological) stimuli.

### 3.1. pH-Responsive Fibers

The pH levels of human tissues and organs vary significantly throughout the body and play critical roles in maintaining normal physiological functions [132]. For instance, healthy human skin typically maintains a pH range of 4.0–5.5 [133]. However, in the case of infected chronic wounds, the pH range can shift from 7.2 to 8.9 [134]. This imbalance has opened avenues for drug delivery strategies [132]. For instance, nanofiber-based pH-responsive delivery systems have been proposed to carry and direct therapeutic agents to specific sites in the body, modulating the release rate of active compounds as pH varies physiologically or pathophysiologically [104,105,107,110,135]. Such drug delivery systems primarily utilize macromolecules that possess pH-dependent functional groups along their backbones, including acidic (such as carboxylic acids) or basic groups (such as primary, secondary, or tertiary amines). The ionization state of these groups changes in response to alterations in pH, thereby modifying the structure of the macromolecular chains and facilitating the release of active compounds [51,135,136]. Nanofibers derived from macromolecules containing carboxylic acid groups, such as poly(acrylic acid) (PAA) [107], demonstrate pH-responsive release of cargoes or drugs, as the electrostatic interaction with the drugs is reduced in an acidic environment. Similarly, nanofibers composed of macromolecules containing amine groups, such as chitosan [23], tend to swell or dissolve, leading to drug release under weakly acidic conditions.

**Figure 3:**
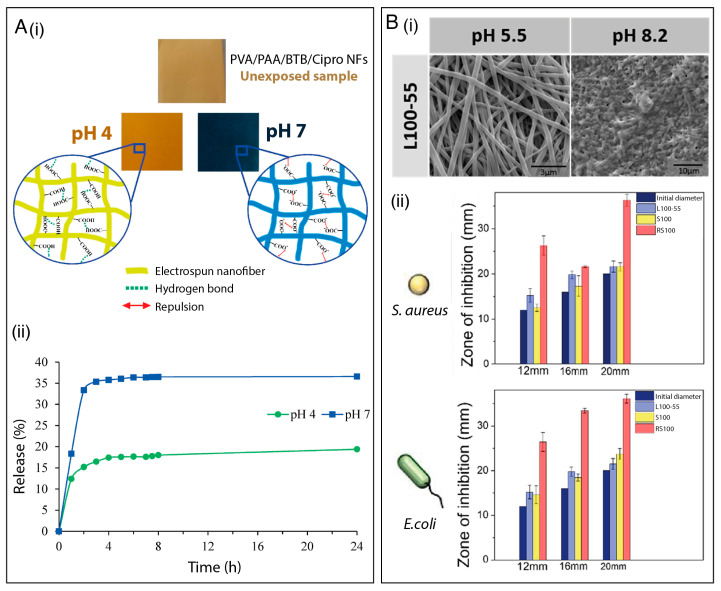
(**A**) (i) Schematic illustration of hydrogen bonding and anion repulsion and color transitions of PVA/PAA/BTB/ciprofloxacin nanofibrous mats under different pH conditions. (ii) Release profile of ciprofloxacin at pH 4 and pH 7. Reprinted with permission from reference [108]. Copyright 2021 Elsevier. (**B**) (i) SEM images of Eudragit L100-55 electrospun nanofibers under different pH conditions. (ii) Antibacterial activity of L100-55/chlorhexidine (CHXD), Eudragit S100/CHXD, and Eudragit RS100/CHXD against *S. aureus* and *E. coli* after 24 h. Reprinted with permission from reference [105]. Copyright 2022 Elsevier.

In one example of such work, polyvinyl alcohol (PVA) and PAA nanofibrous mats were prepared using electrospinning, as depicted in Figure 3A. The mats were loaded with a pH-responsive dye, bromothymol blue (BTB), and an antibacterial drug, ciprofloxacin [107]. The goal was to leverage the pH-responsive properties of PAA and the color-changing properties of BTB to functionalize the mats for on-demand ciprofloxacin release and wound condition monitoring, respectively. The nanofibrous mat underwent a color change from yellowish to green and blue hues when exposed to wound conditions simulating pH levels of 7 and 8.5 (Figure 3A(i)). This change indicates the suitability of the mats for monitoring wound conditions. Furthermore, the maximum drug release increased from 19% to 36% when the pH was increased from 4 to 7 (Figure 3A(ii)). This behavior was attributed to the ionization of the carboxylic acid (–COOH) groups in PAA at higher pH, leading to polymer dissolution and contributing to the faster release of the loaded drug. Moreover, the fabricated electrospun mats demonstrated antibacterial activity against *S. aureus* and *E. coli*.

Boda et al. [104] developed chitosan-based electrospun membranes for the pH-responsive delivery of antimicrobial peptides. In their study, ca. 60%, 40%, and 20% of the loaded peptide was released, at pH 4.5, 5.5, and 6.5 respectively. This arises due to the greater extent of protonation of both the peptide and the amino groups in chitosan with a decrease in pH, and the subsequent faster dissolution of both the polymer and drug cargo. The authors also demonstrated that the higher peptide release rate resulted in more pronounced antimicrobial activity toward oral streptococci bacterial strains (*S. gordonii* and *S. mutans*). In another study, researchers investigated the incorporation of antiviral drugs into electrospun cellulose acetate phthalate (CAP) fibers [110]. These fibers demonstrated remarkable stability in healthy vaginal fluid with a pH below 4.5. However, upon introducing a small amount of human semen with a pH ranging from 7.4 to 8.4, the membranes quickly dissolved, triggering the release of encapsulated drugs. The dissolution mechanism of the fibers could be attributed to the ionization of phthalate groups within the CAP polymeric chains, which occurs at a pH above 5, resulting in the solubilization of the polymer. This finding highlights the potential of CAP-based fibers to effectively prevent the transmission of the human immunodeficiency virus.

Recently, pH-sensitive electrospun wound-dressing membranes incorporating the antiseptic chlorhexidine, the antibiotic rifampicin, and the natural antimicrobial thymol were successfully developed [105]. The dressings were prepared by loading the aforementioned drugs (separately) into three distinct polymer layers. The polymers consisted of the pH-dependent methacrylic acid copolymer Eudragit^®^ L100-55, which dissolves when exposed to pH levels above 5.5, Eudragit^®^ S100, which dissolves at pH levels above 7, and the methacrylic ester copolymer Eudragit^®^ RS100, which slowly erodes and gradually releases active ingredients. The objective was to precisely fine-tune the release kinetics of antimicrobial agents based on the specific pH requirements of individual wounds in a timely and effective manner. Drug release studies were performed by immersing the developed membranes in different pH-buffered solutions (pH = 5.5, 7.4, and 8.2) for 24 h, and the results revealed that the pH of the medium modulated the release rate of the active compound. For instance, at pH 7.4 and 8.2, rifampicin was completely released from the nanofibers due to the dissolution of the Eudragit^®^ L100-55. In contrast, only 25% of this antibiotic was released at pH 5.5, as illustrated in Figure 3B(i). The drug delivery platform exhibited antimicrobial activity against the gram-positive methicillin-sensitive *S. aureus* strain and the gram-negative *E. coli* strain (Figure 3B(ii)). The results demonstrated that combined electrospun layers can be designed to adapt to the acidic or alkaline status of different wounds, thereby facilitating appropriate wound management.

Although promising, the design of pH-sensitive electrospun membranes exhibiting antimicrobial activity is challenging, and these constructs have not been fully explored. Several improvements can still be made to the current electrospinning methods for manufacturing pH-sensitive nanofibers at large and industrial scales. Furthermore, efforts should be made to enable the electrospinning of polyelectrolyte macromolecules commonly used to design pH-sensitive antimicrobial systems. Research efforts should also be devoted to the electrospinning of alternative macromolecules exhibiting acid-labile bonds, including imine (e.g., poly(ethylene glycol)-cholic acid grafted poly-L-lysine (PEG-PLL-CA)), hydrazone (e.g., *N*-(2-hydroxypropyl)methylacrylamide (HPMA)), acetal (e.g., poly(acetal carbamate)), and orthoester (e.g., poly(γ-benzyl L-glutamate) (PBLG)) groups for the design of electrospun pH-sensitive systems [135]. In this case, the release of active compounds can be modulated by the degradation of the acid-labile bonds in a weakly acidic environment. Additionally, there is more progress to be made regarding the application of antimicrobial pH-sensitivity electrospun membranes in other fields besides biomedical applications, such as food packaging and water treatment. Further, additional in vivo studies should be conducted for biomedical applications to improve the comprehension of pH-responsive nanofibrous systems.

### 3.2. Thermo-Responsive Fibers

Since the local temperature difference between infected wounds and normal skin can be as high as 4–5 °C, this thermal difference can be exploited to achieve a triggered release of antimicrobial agents upon a thermal stimulus [137]. Thermo-responsive or temperature-responsive polymers are smart materials that undergo a phase transition, from the open coil state (soluble) to the globule (insoluble) state, in response to temperature changes [26,33,34,138]. Such polymers can be classified into lower critical solution temperature (LCST)-type and upper critical solution temperature (UCST)-type polymers. UCST polymers are non-soluble at low temperatures and become soluble above the UCST. In contrast, LCST-type polymers interact well with their solvent at low temperatures but become dehydrated and adopt a folded structure above their LCST [139]. Both LCST and UCST systems can be strongly affected by several factors, such as polymer concentration, molecular weight, and the presence of surfactants, salts, or other solvents in the system, which opens the way to finely tune the temperature range over which a response occurs [140].

**Figure 4:**
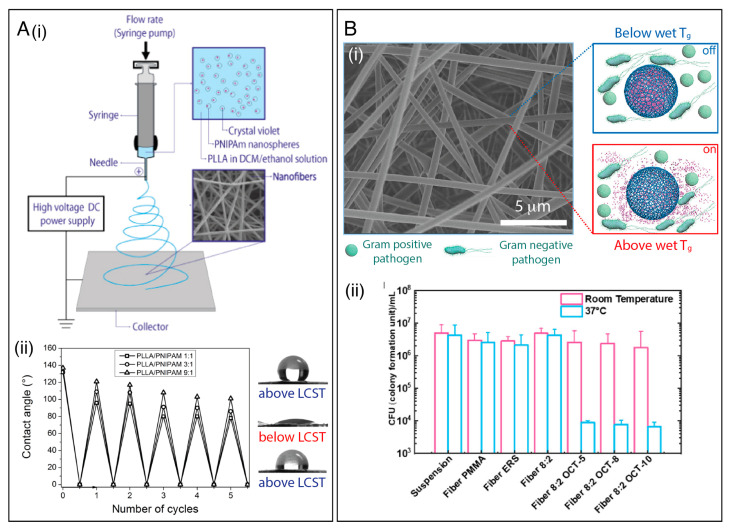
(**A**) (i) Schematic illustration of the process of encapsulating crystal violet (CV) into PLLA/PNIPAam nanofibrous membranes. (ii) Temperature-governed wettability switching cycles of the nanofibrous membrane with different polymer ratios, with representative photos of droplets on the membrane after preparation (above LCST, top), before fully flowing out (below LCST), and after five cycles of switching (above LCST, bottom). Reprinted with permission from reference [144]. Copyright 2017 Elsevier. (**B**) (i) Schematic illustration of T_g_-triggered octenidine (OCT) release from the ERS/PMMA electrospun fibers. By switching the temperature to above or below the T_g_, a pulse-like OCT release from the fibers can be regulated and used to optimize a controlled antimicrobial dosage. (ii) Antimicrobial analysis of OCT-loaded fibers against *P. aeruginosa* at room temperature and at 37 °C. Reprinted with permission from reference [36]. Copyright 2021 American Chemical Society.

Thermosensitive polymers often contain hydrophilic and hydrophobic domains, either by incorporating monomers presenting both characteristics or using mixtures of monomers presenting varying structures/functionalities [20]. The main classes of LCST-type polymers are poly(*N*-alkyl substituted acrylamides), having transition temperatures around 32–40 °C which are dependent upon chain length [139]. Among them, poly(*N*-iso-propylacrylamide) (PNIPAAm) is one of the most studied and applied thermo-sensitive polymers, with an LCST of about 32 °C. However, its electrospinnability is limited [141]. An easy and effective approach to overcome this issue is to blend it with an easily electrospinnable polymer, such as polycaprolactone (PCL) [142], poly(L-lactide) (PLLA) [112,141], poly(methyl methacrylate) (PMMA) [143,144], and PVDF [145]. In this direction, Elashnikov et al. [112] prepared a thermo-responsive nanofibrous membrane by electrospinning PLLA, PNIPAAm nanospheres, and crystal violet (CV) as a model antibacterial agent (Figure 4A(i)). As shown in Figure 4A(ii), the nanofibrous membrane exhibited a reversible switch between superhydrophilicity and hydrophobicity when the temperature varied between 24 (below LCST) and 37 °C (above LCST), leading to different CV release profiles as a function of the PNIPAAm nanosphere concentration. Moreover, the interaction between PNIPAAm and PLLA affected the PNIPAAm phase transition temperature, which shifted to a lower value with an increase in PLLA content. The temperature-responsive release also governed the antibacterial activity of the membrane, which was investigated against *Staphylococcus epidermidis* (*S. epidermidis*) and *E. coli*. The authors observed that the inhibition zone decreased with a decrease in the PNIPAAm content due to lower CV release. Moreover, the antibacterial activity was more significant at temperatures below the LCST, where the PNIPAAm was swollen.

Other examples of LCST polymers include poly(*N*,*N*-diethylacrylamide) (PDEA, LCST = 25–32 °C), poly(*N*-vinylcaprolactam) (PVCL, LCST = 25–35 °C), poly(2-(dimethylamino)ethyl methacrylate) (PDMAEMA, LCST~50 °C), and poly(ethylene oxide) (PEO, LCST = 100–180 °C) [34,50]. PEG analogs based on 2-(2-methoxyethoxy)ethyl methacrylate (MEO2MA), oligo(ethylene glycol) methacrylate (OEGMA), 2-hydroxyethyl methacrylate (HEMA) copolymers, and the poly(ethylene glycol)-poly(propylene glycol)-poly(ethylene glycol) (PEG–PPG–PEG) triblock copolymer have also been electrospun to develop thermo-responsive systems [146]. For instance, Pan and coworkers [36] reported that a polymer or polymer blend’s glass transition temperature (T_g_) could be exploited to achieve thermal-triggered antimicrobial drug release scaffolds at the physiological temperature, as illustrated in Figure 4B(i). In their work, octenidine (OCT), an antimicrobial agent often used in wound treatment, was encapsulated into Eudragit^®^ RS 100 (ERS)/PMMA nanofibers. By varying the ERS/PMMA blending ratio, the OCT releases could be regulated by the temperature change from 25 to 37 °C. The nanofibrous membranes displayed antibacterial activity, causing a reduction in the viable cells of both Gram-negative *P. aeruginosa* (Figure 4B(ii)) and Gram-positive *S. aureus* pathogens at 37 °C. Additionally, the “on/off” thermal switch for controlled drug release could be repeated at least five times.

Thermo-responsive shape memory polymers, which can change shape on demand in response to an environmental stimulus, have also been used to develop smart materials. These smart materials generally contain a cross-link, which determines the permanent shape, and a thermally reversible phase for maintaining the temporary form below the switching temperature [147]. In this direction, Yin et al. [114] explored this strategy to release a natural antibacterial agent, berberine hydrochloride (BCH), from a polyurethane shape memory (SMPU) nanofibrous membrane. The bottom and top layers were fabricated by electrospinning a SMPU solution, and the inner drug-loaded layer was made from an SMPU/BCH mixed solution. The authors demonstrated that the BCH could be released in a controlled manner owing to the thermo-sensitive shape memory effect, and the release rate of BCH could be accelerated by stretching and fixing the nanofibrous membranes into specific ratios before release. A larger deformation ratio led to a faster release rate due to the reduced diffusion pathway, and the membrane showed good antibacterial activity against *E. coli* and *S. aureus*.

### 3.3. Light-Responsive Fibers

Light-responsive materials have shown unique advantages for designing smart antimicrobial systems by tuning light parameters, such as wavelength, irradiation intensity, and exposure duration [148]. Photoresponsive systems generally use ultraviolet (UV) (200–400 nm), visible (Vis; 400–700 nm), and near-infrared (NIR; 700–1000 nm) as radiation sources [149]. Light-responsive antibacterial materials usually consist of organic molecules (e.g., chromophores or fluorophores) or inorganic nanomaterials (e.g., carbon-based, metal, and metal oxide nanostructures), which can be used as agents for photodynamic therapy (PDT), photothermal therapy (PTT), or drug delivery [75,148].

**Figure 5:**
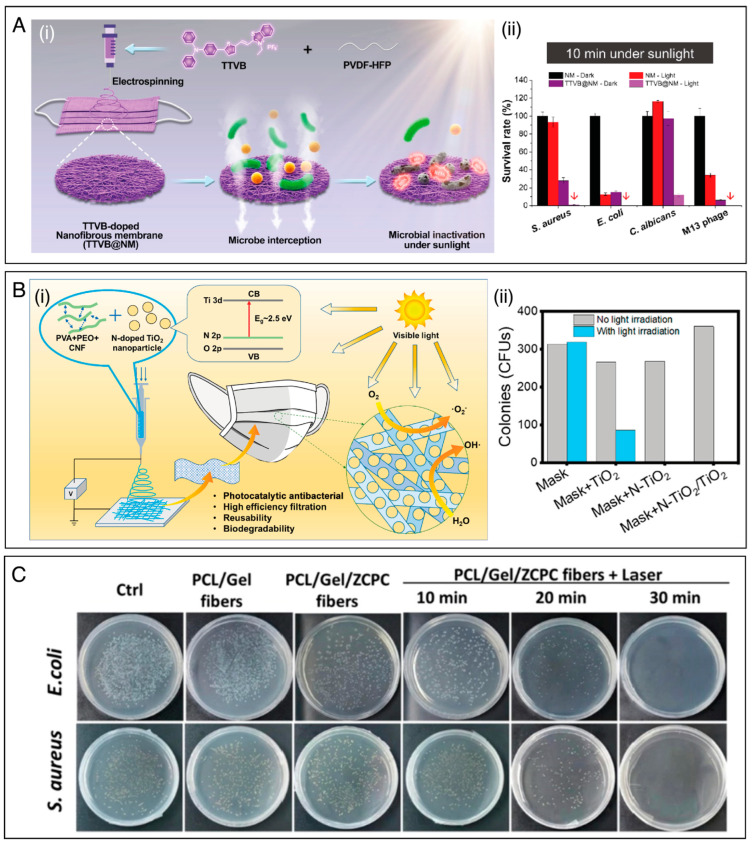
(**A**) (i) Schematic representation of the preparation of TTVB@NM electrospun membrane for microbe interception and microbial inactivation under sunlight. (ii) Survival rates of different microbes after 10 min under sunlight irradiation. Reprinted with permission from reference [116]. Copyright 2021 Elsevier. (**B**) (i) Schematic illustration of the functional and structural design of PVA/PEO/nanocellulose electrospun membranes functionalized with photocatalytic TiO_2_. (ii) The CFU counts on the plates before and after light irradiation. Reprinted with permission from reference [37]. Copyright 2021 American Chemical Society. (**C**) Agar plate digital photographs of *E. coli* and *S. aureus* treated under different conditions, including the use of PCL/gelatin (Gel) nanofibers and a functionalized NIR-triggered nanoagent (ZCPC). Reprinted with permission from reference [123]. Copyright 2022 Elsevier.

The mechanism of PDT is dependent on the generation of photocatalytic ROSs, which most commonly comprise superoxide anions (•O_2_^−^), hydrogen peroxide (H_2_O_2_), singlet oxygen (^1^O_2_), and hydroxyl radicals (•OH) [54]. For example, Li et al. [116] incorporated an organic photosensitizer (TTVB) into a poly(vinylidene fluoride-co-hexafluoropropylene) (PVDF-HFP) solution and processed this by electrospinning to obtain a nanofibrous membrane (TTVB@NM) for microbe interception and microbial inactivation under sunlight (Figure 5A(i)). Upon sunlight irradiation, TTVB@NM showed a high inactivation rate against several pathogens, including *S. aureus*, *E. coli*, *Candida albicans* (*C. albicans*), and the M13 bacteriophage vector, as shown in Figure 5A(ii). The effect of ROSs in viral decontamination for face masks has been investigated by Shen et al. [122]. In their work, electrospun nanofibrous membranes were prepared by electrospinning PVDF and rose bengal. The membranes showed excellent performances in capturing and deactivating murine hepatitis virus A59 (MHV-A59), a coronavirus surrogate for SARS-CoV-2. Specifically, the membrane removed almost 100% of MHV-A59 aerosols and inactivated around 97% of MHV-A59 droplets after only 15 min of desk lamp irradiation.

The irradiation of photocatalyst materials (e.g., transition metal oxides, carbon-based nanomaterials) with light, of energy equal to or greater than their band gap, can promote the excitation of electrons from the valence band to the conductive band, thus resulting in the formation of excited electron–hole pairs. The interaction between excited electron–hole pairs and ambient air molecules through the oxidation and reduction processes induce the generation of ROSs, enabling the use of photocatalysts in PDT [43,46,150]. For instance, TiO_2_ has been considered a promising material for PDT. However, its wide optical band gap (3.2 eV) may restrict its applications in clinical areas, since UV has a limited penetration depth through the skin [151]. To overcome this issue, the doping strategy appears to be an efficient approach to enhance interfacial charge transfer and restrict electron–hole recombination, improving the photocatalytic activity of TiO_2_ under visible light. Li et al. [37] designed a nanofibrous antibacterial mask based on polyvinyl alcohol (PVA), PEO, and cellulose (CNF) decorated with nitrogen-doped TiO_2_ (N–TiO_2_) and TiO_2_ nanoparticles (NPs) (Figure 5B(i)). By doping the TiO_2_ NPs with nitrogen, the energy band gap of the TiO_2_ decreased to 2.77 eV, thus resulting in a red-shift in the absorption spectrum. The as-prepared N–TiO_2_/TiO_2_ mask revealed 100% effective bacteria sterilization against *E. coli* and *S. aureus* under either sun-simulator irradiation (200–2500 nm, 106 W m^−2^) or natural sunlight for 10 min. Moreover, the antibacterial activity remained stable after three sterilization cycles, without significantly decreasing filtration efficiency. In contrast, as shown in Figure 5B(ii), most bacteria remained alive on the same membrane without light exposition.

Under light stimulation some materials, including metal, metal oxides, and carbon-based materials, can convert solar energy into heat, which can also kill pathogenic bacteria through a PTT mechanism [148]. For example, Tian et al. [27] reported an efficient antimicrobial PTT system based on an Au@carbon dots (Au@CDs) composit embedded within a poly(vinyl alcohol) (PVA) nanofibrous membrane. Thermal infrared images showed that the Au@CD/PVA membrane exhibited excellent photothermal conversion ability under 808 nm NIR laser irradiation for 10 min. Specifically, when laser irradiation at 3 W⋅cm^−2^ was used, the Au@CDs/PVA membrane exhibited a much more significant temperature increase (to 50–64 °C) when compared to the control samples (PVA and PVA/Au membranes). In vitro photothermal antibacterial inactivation studies confirmed the composite membrane’s efficacy against *S. aureus* and *E. coli* (99% inactivation under NIR irradiation). The system also showed low cytotoxicity, which should act to promote the healing of infected wounds.

In other work, Chen et al. [123] designed a photothermal-responsive fiber dressing consisting of aligned PCL/gelatin (Gel) nanofibers functionalized with nanocarbons derived from zeolitic imidazolate framework-8 (ZIF-8). The nanocarbons were mixed with ciprofloxacin hydrochloride (CIP) and sodium polyacrylate (PAAS) to create a NIR-triggered membrane (ZCPC). Due to photothermal-triggered melting of the PCL/Gel fibers, NIR light irradiation could induce the release of CIP and Zn^2+^ ions (present in ZIF8) for rapid bacterial eradication, increasing antibacterial efficacy to more than 98% for *E. coli* and *S. aureus* (Figure 5C). The results showed that combining release and local heating is useful for improving antibacterial efficacy and achieving bacterial elimination. It could also promote the healing of bacteria-infected wounds in mice. In another study, Ballesteros and coworkers [152] developed a smart PCL nanofibrous mat decorated with photoresponsive nanogels containing silver nanoparticles (AgNPs). Due to their specific surface plasmon resonance effect, the AgNPs converted absorbed light into heat energy, breaking the nanogels and simultaneously releasing a large amount of silver NPs. Inhibition zone diameter evaluation evidenced the broad-spectrum antibacterial property of the as-prepared PCL/AgNPs-nanogels against *S. aureus* and *E. coli*.

### 3.4. Other Types of Stimuli

Besides responding to pH, light, and temperature, other types of stimuli can successfully trigger antimicrobial activity in a system. These stimuli include acoustic fields, electrical fields, or even biological mechanisms involving the body’s defense cells.

Ultrasound-targeted drug delivery comprises a safe mechanism involving the use of acoustic irradiation during treatment. This can facilitate drug diffusion between cells in the processes of tissue regeneration, for instance [153]. The ultrasound technique permits high spatial and temporal resolution [153,154] and can also be used for medical application for local antimicrobial delivery [77]. For instance, Khorshidi and Karkhaneh [77] demonstrated the release of ciprofloxacin hydrochloride (CipHCl) encapsulated into alginate/PEO nanofibers. Under cycles of 10 min of acoustic stimulation (15 W/cm^2^), antibiotic delivery increased more than three times compared to mats that did not receive ultrasound stimulation, with a superior antibacterial activity. The acoustic field temporarily perturbates the ionic crosslinks between alginate molecules in the fibers, and thus causes the release of the antibiotic.

Wearable pressure sensors with antibacterial properties based on electrospun nanofibers containing piezoelectric materials have also been developed by many research groups [155,156,157,158,159,160]. For instance, a pressure sensor with good antibacterial performances was fabricated based on electrospun PVDF incorporating Ba(Ti_0.8_Zr_0.2_)O_3_-0.5(Ba_0.7_Ca_0.3_)TiO_3_ (PVDF/BZT-0.5BCT NFs) [161]. The pristine PVDF NFs exhibited little antimicrobial activity, while the PVDF/BZT-0.5BCT NFs showed good antibacterial activity against both *E. coli* and *S. aureus*. Specifically, when the ultrasonic exposure time reached 60 min, the antibacterial rates reached 99.9% and 98.4% against *E. coli* and *S. aureus*, respectively. This behavior was attributed to the fact that the BZT-0.5BCT NPs could generate a piezoelectric potential under ultrasound vibration, which could further promote the generation of ROSs.

Recent works have proposed novel concepts of antibiotic release triggered by the immunological response, with no external stimulation required [36,126]. For instance, this could be triggered by the enzyme cholesterol esterase (CE), which is secreted by the macrophages accumulated in a wound site and is able to catalyze the degradation of some polymers containing esters [162]. An interesting infection-responsive nanofibrous mat presented by Shi et al. [126] could trigger antibiotic release as soon as the body’s infection response is activated. For this, PCL nanofibers were coated with polydopamine (PDA) functionalized with siloxane, providing amino groups permitting the covalent attachment of the antibiotic metronidazole (MNA) via ester linkages. These ester linkages can be hydrolyzed by CE, and the platform could thus provide an infection-stimulated release of MNA. The bacteriostatic efficacy of the modified PCL nanofibrous mat was sensitive to the concentration of CE. Compared to control groups without CE stimulation, the bioresponsive material could reduce *Helicobacter pylori* viability to as little as 34%.

Electrochemically controlled drug delivery systems have been studied since the 1980s, and their use in clinical applications has been shown in recent works aiming at wireless, self-powered, systems and precision medicine. The mechanisms involved in release can be related to alterations in the redox state [163], electrostatic interactions [164], electrophoresis, and iontophoresis [165], among others. For instance, nanofibrous mats composed of PLA and graphene oxide (GO) were used to encapsulate the natural flavonoid quercetin [124]. The in vitro release of quercetin was significantly faster when an electrical stimulus was applied and the GO content was increased, resulting in an antibacterial effect against *S. aureus*, *E. coli*, and *C. albicans*. Bacteriostatic effects ranging between 41 to 76% were observed, followed by inhibition of bacterial film growth.

**Figure 6:**
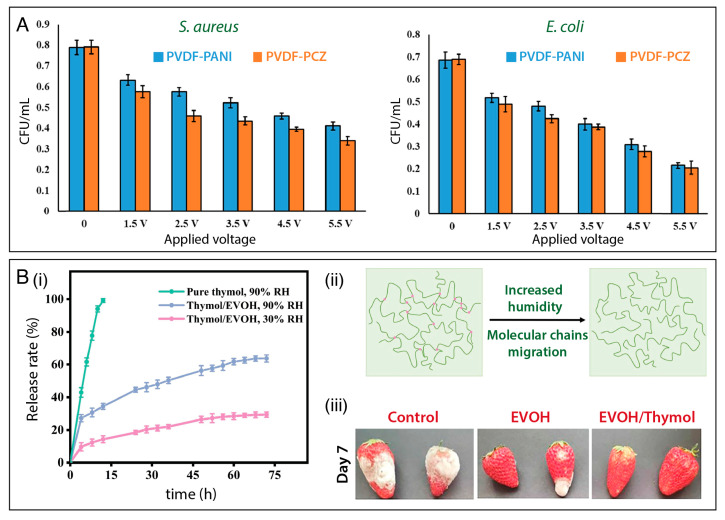
(**A**) Bacterial inhibition activity against *S. aureus* and *E. coli* under electrical stimulation of PVDF/PANI, and PVDF/PCZ nanofibrous membranes. Reprinted with permission from reference [168]. Copyright 2021 American Chemical Society. (**B**) (i) Cumulative release profiles of pure thymol at 90% RH and thymol encapsulated in a EVOH nanofibrous mat at 30% RH and at 90% RH. (ii) Schematic illustrations of EVOH molecular chain migration under humidity. (iii) Data showing the potential of using thymol-loaded EVOH membranes for preserving strawberries. Reprinted with permission from reference [125]. Copyright 2022 Elsevier.

To boost the resistance of wearable devices against bacterial fouling arising from usage and contact with body fluids, Sengupta and coworkers [166] reported the design of a piezoelectric and energy harvesting nanofibrous platform based on PVDF blended with polycarbazole (PCZ) or polyaniline (PANI). As shown in Figure 6A, a notable decrease in *E. coli* and *S. aureus* growth was observed as the PVDF/PCZ and PVDF-PANI nanofibers were subjected to an increasing electrical stimulus (up to 5.5 V), leading to a CFU decrease as low as 50% for *S. aureus* and 30% for *E. coli*.

Humidity-responsive materials have also attracted increasing attention for the controlled release of antimicrobial agents [167]. Zhang et al. [125] established a plant leaf-stomata-inspired packaging film to trigger thymol release at different relative humidity conditions. Thymol was encapsulated into an ethylene vinyl alcohol copolymer (EVOH) to form core–shell nanofibers via coaxial electrospinning. The authors showed that the thymol/EVOH membrane could regulate the thymol release by adjusting the RH. As shown in Figure 6B(i), the nanofibers released markedly more thymol at 90% than at 30% RH, due to the increased chain mobility of EVOH as the RH increased, which enhanced the rate of thymol diffusion (Figure 6B(ii)). The membrane showed excellent antibacterial activity in vitro against *E. coli* and *S. aureus*. Moreover, the results suggested that the membrane could act as a protective packaging material for strawberries (Figure 6B(iii)), enhancing resistance against fungi.

### 3.5. Stimuli-Responsive Antimicrobial Systems Based on Combined Approaches

Stimuli-responsive antimicrobial systems based on combined approaches allow one or more therapeutic agents to be loaded into a carrier and systematically released in the presence of a specific stimulus [116,130,168]. Such a strategy offers advantages such as (i) amplification of antibacterial spectrum of action; (ii) ability to act over polymicrobial infections; (iii) synergism between actions of compounds or therapies, resulting in greater antimicrobial effects; and (iii) decreased chance of developing multidrug bacterial resistance [4,8]. A number of studies have demonstrated the antimicrobial effects of using such strategy, as described in Table 1.

Abdalkarim et al. [130] reported a thermo- and light-responsive phase change nanofiber (PCF) based on 3-hydroxybutyrate-co-3-hydroxy valerate (PHBV) functionalized with polyethylene glycol (PEG) and cellulose nanocrystal-zinc oxide (f-CNC-ZnO) nanohybrids. PHBV is a biodegradable polymer widely applied in the biomedical field; however, some of its properties (e.g., hydrophobicity, crystallinity, brittleness, and poor thermal and mechanical properties) need to be modulated by the addition of other components. To this end, a cellulose nanocrystal-ZnO nanohybrid was added to provide antimicrobial, mechanical, UV shielding, and thermal stability at elevated temperatures, while PEG was added as a phase change material. Tetracycline hydrochloride (TH) was encapsulated in this material during the electrospinning process, alongside other components. The authors observed that below the melting point of PEG (60 °C), TH release was minimal, attributed to the slow diffusion of the antibiotic within the solid matrix. However, once the temperature surpassed the melting point of PEG, the encapsulated TH could be readily liberated from the molten PCF composite.

**Figure 7:**
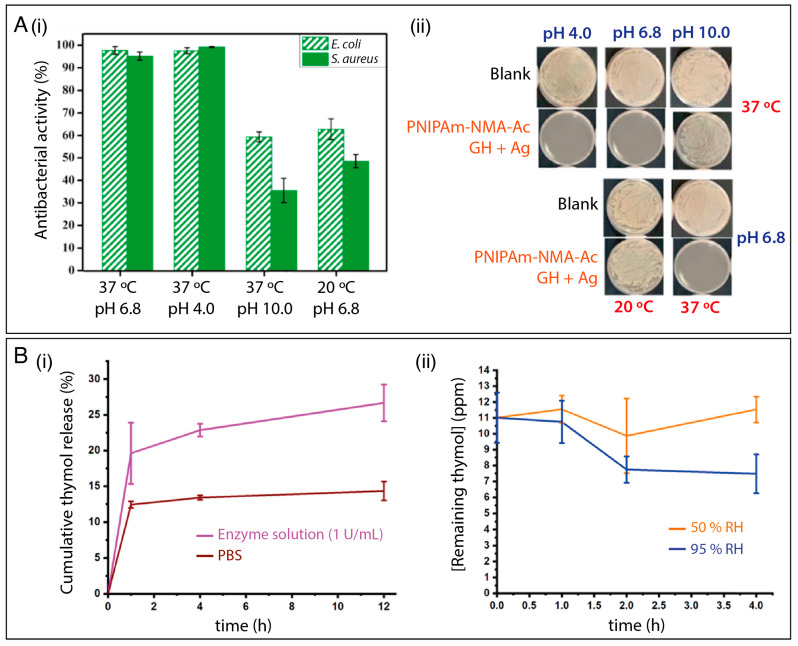
(**A**) (i) The antibacterial activity of the PNIPAm-NMA-Ac GH + Ag fibrous membrane under different environments and (ii) optical images of experiments probing the effect of the formulation on *E. coli* growth. Reprinted with permission from reference [53]. Copyright 2021 Elsevier. (**B**) (i) Cumulative release of thymol from enzyme-responsive fibers and (ii) remaining thymol concentration in the fibers at 50% and 95% RH. Reprinted with permission from reference [127]. Copyright 2021 American Chemical Society.

Wei et al. [53] reported the design of a pH- and thermo-responsive system by the incorporation of AgNPs and gatifloxacin hydrochloride (GH) into poly(*N*-isopropyl acrylamide-*N*-methylol acrylamide-acrylic acid) (PNIPAm-NMA-Ac) nanofibers. The cumulative release of Ag and GH was investigated at 20 °C and 37 °C, and at pH 4, 6.8, and 10. The authors observed that release was notably higher at 37 °C/pH 4 than at 37 °C/pH 10 (Figure 7A(i)). Antibacterial experiments (Figure 7A(ii)) demonstrated that the PNIPAmNMA-Ac GH + Ag demonstrated more evident antibacterial activity than the platforms containing GH and Ag separately. Lower antibacterial activity against *E. coli* and *S. aureus* was seen at 20 °C and pH 10, in accordance with the drug release data.

Multi-stimuli-responsive systems can also be applied to design active packaging, as exemplified by a platform based on CNC, zein, and starch nanofibers [127]. A cocktail of antimicrobial compounds, including thyme oil, citric acid and sorbic acid, and cyclodextrin-inclusion complexes were added to the polymer solution before the electrospinning process. The cyclodextrin–drug complexes can be disassociated by weakened hydrogen bonds when the RH exceeds 85%. Release studies were investigated in neat PBS (pH 7.4), PBS buffered enzymes solutions (protease, amylase and cellulose) at 0.1, 1, and 3 U/mL, at 50% and 95% RH at 22 °C. As shown in Figure 7B(i), active ingredient release in the presence of the enzymes was increased. The authors highlighted that the release mechanism in PBS is mainly governed by diffusion, but in the presence of the enzymes, the thymol is released both by diffusion and by degradation of the cellulose, zein, and starch. Additionally, an increase in the rate of release was seen under higher RH conditions, owing to the dissociation of the cyclodextrin complexes. In another work, an antimicrobial system responsive to three separate stimuli was developed by Gorji et al. [131] using a curcumin-loaded electrospun membrane of poly(methyl methacrylate)-co-poly(*N*,*N*-diethylaminoethyl methacrylate) (PMMA-co-PDEAEMA). This delivers curcumin when stimulated by electric potential, CO_2_ gas, and the pH of the surrounding medium. The curcumin-release mechanism appeared to be associated with the membrane’s controllable hydrophilicity.

## 4. Final Remarks and Future Perspectives

In this review, we have surveyed several types of stimuli-responsive antimicrobial materials prepared using the electrospinning technique. Our review shows that stimuli-responsive nanofibrous membranes hold immense promise in antimicrobial research in the coming decades.

Despite the considerable progress achieved so far, there exist both challenges and opportunities for further development. As showed in Table 1, most reported studies focus on a small list of common stimuli-responsive materials. Therefore, more research is required to expand the list of smart materials for electrospinning. Moreover, effort is needed to understand the relationships between the fiber structures and the resultant stimulus-responsive behavior, allowing the rational design of membranes with exceptional antimicrobial performance. This advance will involve selecting the polymer, the type of architecture to be created (simple, core-shell, Janus, or sandwich, etc.), and which organic and/or inorganic compounds should be incorporated into these materials to optimize the synergy between structure, performance, processing, and desired properties for a given application. Furthermore, there is also a tendency for the development of wearable, intelligent, responsive, multifunctional, and antimicrobial materials, capable of monitoring human health [49,169,170,171,172]. Moreover, the combined use of electrospinning and 3D printing is expected to increase in the next years, enabling the construction of responsive, intelligent, multifunctional, and antimicrobial nanostructured materials with more complex, personalized, and customized designs [99,100,101,173,174].

The key requirement for future work is a focus on scale: to date, the vast majority of research is limited to lab-scale exploration and proof-of-concept studies on model systems. Therefore, it is crucial to identify suitable scenarios for real-world applications of stimuli-responsive nanofibers. Looking further forward, exploring scaled-up fabrication, expanding manufacturing capabilities, and the durability/stability of stimuli-responsive nanofibrous mats manufactured in large areas must be investigated and improved. By addressing these criteria, and through continued innovation, integrating stimuli-responsiveness into electrospun nanofibers offers promising opportunities in membrane science for novel biomaterials, tissue engineering, drug delivery, wound dressings, food packaging, and filtration.

## Figures and Tables

**Table 1:** Examples of stimuli-responsive electrospun nanofibers with antimicrobial properties.

Stimuli	Nanofibers Matrix	Antimicrobial Agent	Microorganism	Observations	Ref.
pH	Chi/Pect/HPγCD	Curcumin (polyphenol)	-	Nanofibers exhibited a pH-responsive release profile of curcumin in pH 5.4 and 7.4.	[35]
	Chi/oxidized pectin	D-GL13K or IDR-1018 (peptides)	*S. gordonii*, *S. mutans*	Oxidized pectin aid in the pH-controlled delivery of cationic peptides.	[104]
	Eudragit^®^ L100-55 or Eudragit^®^ S100 or Eudragit^®^ RS100	Thymol (monoterpene) Rifampicin (antibiotic)	*E. coli*, *S. aureus*	Varying the polymeric matrix of nanofibrous membranes enabled different antimicrobial release kinetics.	[105]
	PDMA	Amoxicillin (antibiotic)	*E. coli*, *S. aureus*	The different release profiles observed upon different pH was due to the reversible protonation and deprotonation of side-chain tertiary amine groups in PDMA.	[106]
	PVA/PAA/BTB	Ciprofloxacin (antibiotic)	*E. coli*, *S. aureus*	The ionization of –COOH groups of PAA at higher pH (>7) causes repulsion among -COO^-^ enhancing the drug release.	[107]
	CNF-PEI	-	*E. coli*, *L. monocytogenes*	The protonation and deprotonation of NH_2_ groups and the competition of intermolecular hydrogen bonds in CNF-PEI as a function of pH led to a reversible change of the wettability and antibacterial properties.	[108]
	PD-ChNFs	-	*E. coli*	The antibacterial effect is caused by the cationic amino group content that was affected by degree of deacetylation and pH.	[109]
	CAP	Reverse transcriptase inhibitors TMC 125 or tenofovir disoproxil fumarate (Viread)	BAL virus	CAP fibers rapidly dissolve at pH 7.4, releasing the encapsulated drugs.	[110]
Temperature	PNIPAM/PVA	ZnO	*S. aureus*	Nanofibers exhibited a thermo-controllable ZnO release profile upon temperature variation between 28 and 32 °C.	[111]
	Eudragit^®^ RS 100/PMMA	Octenidine dihydrochloride (antiseptic)	*S. aureus*, *P. aeruginosa*	The thermal switch can be turned on at 37 °C and off at 25 °C, conferring a controlled release of the antiseptic.	[36]
	PLLA/PNIPAM	Crystal violet (non-toxic dye)	*E. coli*, *S. epidermidis*	Switchable wettability and controlled release were achieved by changing the environmental temperature across the LCST of PNIPAM.	[112]
	PBS	Aminophosphonates derivatives	*E. coli*, *S. aureus*, *C. albicans*, *K. pneumonia*, *B. subtilis*	Nanofibers exhibited biocidal activity against all tested microorganisms at 39 °C (temperature of an infected wound).	[113]
	SMPU	Berberine hydrochloride (alkaloid)	*E. coli*, *S. aureus*	The alkaloid could be released in a controlled manner owing to the thermo-sensitive shape memory effect of the polymer.	[114]
Light	PLA	Indocyanine green (non-toxic dye)	*S. saprophyticus*, *E. coli*, *S. aureus*	Upon laser irradiation at 810 nm for 30 min, the bacterial viability was significantly reduced.	[115]
	PVDF-HFP	AIEgens	*S. aureus*, *E. coli*, *S. cerevisiae*, M13 bacteriophage	Enhanced antimicrobial performance against pathogens was achieved under sunlight irradiation for 5–10 min.	[116]
	PAN	TiO_2_	*E. coli*, *Bacillus* sp.	At light conditions, PAN-TiO_2_ inhibited 3-fold the growth of bacteria compared to PAN after 24 h.	[117]
	PVA/PEO/CNF	N-TiO_2_/TiO_2_	*E. coli*, *S. aureus*	Antibacterial mask reached 100% bacteria disinfection under 0.1 sun simulation or natural sunlight for only 10 min.	[37]
	PCL	AgNPs	*E. coli*, *S. aureus*	Nanofibrous mats functionalized with photoresponsive nanogels released AgNPs when irradiated by light at 405 nm.	[118]
	PMMA/TPP	AgNPs	*S. epidermidis*, *E. faecalis*	Combining TPP and AgNPs enabled light-triggered tuning of AgNPs release from nanofibers.	[119]
	PAN	CQDs	*E. coli*, *S. aureus*, *P. aeruginosa*, *B. subtilis*	^1^O_2_ generated from the CQDs were responsible for the pathogen inactivation.	[120]
	PCL	uCNT@PDA	*E. coli*, *B. subtilis*	The temperature increase triggered by NIR exposure was enough to kill bacteria and destroy bacterial biofilms.	[121]
	PVDF	Rose bengal	Murine hepatitis virus A59 (MHV-A59)	The membranes rapidly inactivated 97.1% of MHV-A59 in virus-laden droplets after 15 min irradiation of simulated reading light.	[122]
	PVA	-	*E. coli*, *S. aureus*	The temperature increase (up to 50 °C) upon NIR irradiation induced by the Au@carbon dots could effectively eradicate bacteria at the wound site.	[27]
	PCL/gelatin	Ciprofloxacin (antibiotic) and Zn^2+^	*E. coli*, *S. aureus*	The composite fiber membrane with NIR-induced hyperthermia and Zn^2+^ release exhibited bacteriostatic properties	[123]
Ultrasound	PEO	Ciprofloxacin (antibiotic)	*E. coli*, *S. aureus*	Ultrasound stimulus of 15 W/cm^2^ increased the antibiotic release more than three times.	[77]
Electrical	PLA/GO	Quercetin (flavonoid)	*E. coli*, *S. aureus*, *C. albicans*	10 s of electric stimulation at 10 and 50 Hz ensured the complete delivery of the quercetin.	[124]
Humidity	EVOH	Thymol (monoterpene)	*E. coli*, *S. aureus*	Nanofibers released more thymol at 90% RH than at 30% RH.	[125]
Enzyme	PCL	Metronidazole (antibiotic)	*H. pylori*	As the concentration of cholesterol esterase (CE) (an enzyme secreted by macrophagocytes that accumulates at the site of infection) increased, a higher amount of the antibiotic was released from the nanofiber mat.	[126]
Enzyme and humidity	Starch/zein/CNC	AIs (thyme oil, citric acid, nisin) and CD-ICs of AIs	*E. coli*, *L. innocua*, *A. fumigatus*	Fibers could release free AIs when triggered by microorganism-exudated enzymes and AIs from CD-IC in response to high relative humidity (95% RH).	[127]
Enzyme and pH	Eudragit S-100	Clarithromycin (antibiotic)	*E. coli*, *S. aureus*, *S. enterica*	The nanofibers released more antibiotic in response to a pathologic (enzyme hyaluronate lyase) and physiologic (pH 7.4) stimulus.	[128]
Temperature and pH	PNIPAm-*co*-Aam/PCL	Ciprofloxacin (antibiotic)	*S. epidermidis*, *E. coli*	LCSTs of the PCL/PNIPAm-*co*-AAm nanofibers were 32 °C for pH 4 and 37.5 °C for pH 7.4, leading to different ciprofloxacin release kinetics.	[129]
Temperature and light	PEG/PHBV/*f*-CNC-ZnO	Tetracycline hydrochloride (antibiotic)	-	Beyond the PEG melting point, the encapsulated antibiotic was readily released from composite nanofibers.	[130]
CO_2_, pH, and electrical	PMMA-*co*-PDEAEMA	Curcumin (polyphenol)	*E. coli*, *S. aureus*	Increasing voltage from 2 to 8 V, bubbling CO_2_ gas and lowering the pH from 7.4 to 5 lead to an enhanced curcumin release.	[131]

Chi: chitosan; Pect: pectin; HPγCD: starch-derived cyclodextrin (hydroxypropyl-γ-cyclo-dextrin); PDMA: poly(N,N′-diethylamino ethyl methacrylate-co-methyl methacrylate-*co*-acryloyl benzophenone); PAA: poly(acrylic acid); BTB: bromothymol blue; PEI: poly(ethyleneimine); PD-ChNFs: partially deacetylated chitin nanofibers; CAP: cellulose acetate phthalate; PLA: poly(lactic acid); PNIPAM: poly(N-isopropylacrylamide); PLLA: poly-L-lactide; LCST: lower critical solution temperature; PBS: polybutylene succinate; SMPU: sensitive polyurethane shape memory; PVDF-HFP: poly(vinylidene fluoride-*co*-hexafluoropropylene); AIEgens: aggregation-induced emission lumiinogens; PAN: polyacrylonitrile; PVA: poly(vinyl alcohol); PEO: poly(ethylene oxide); CNF: cellulose nanofiber; PCL: polycaprolactone; PMMA: poly(methylmethacrylate); TPP: meso-tetraphenylporphyrin; CQDs: carbon quantum dots; uCNT: unzipped carbon nanotubes; PDA: polydopamine; PVDF: poly(vinylidene fluoride); PVA: poly(vinyl alcohol); GO: graphene oxide; EVOH: ethylene vinyl alcohol copolymer; RH: relative humidity; AIs: antimicrobial active ingredients; CD-ICs: cyclodextrin-inclusion complexes; CNC: cellulose nanocrystals; PNIPAm-*co*-Aam: poly(N-isopropylacrylamide-*co*-acrylamide); PEG: poly(yethylene glycol); PHBV: (3-hydroxybutyrate-co-3-hydroxy valerate); *f*-CNC-ZnO: light-responsive functionalized cellulose nanocrystal-zinc oxide; PMMA-*co*-PDEAEMA: poly(methyl methacrylate)-*co*-poly(N,N-diethylaminoethyl methacrylate).
